# Exploring potential sex differences in Hirschsprung disease: a national cohort study of diagnostic patterns and early postoperative outcome

**DOI:** 10.1007/s00383-025-06213-5

**Published:** 2025-10-03

**Authors:** Linnea Söderström, Christina Graneli, Kristine Hagelsteen, Anna Gunnarsdottir, Jenny Oddsberg, Pär-Johan Svensson, Helena Borg, Matilda Bräutigam, Elisabet Gustafson, Anna Löf Granström, Pernilla Stenström, Tomas Wester

**Affiliations:** 1 Department of Women’s and Children’s Health, Karolinska Institutet, Stockholm, Sweden; 2 Department of Pediatric Surgery, Skåne University Hospital Lund, Lund, Sweden; 3 Department of Pediatrics, Clinical Sciences, Lund University, Lund, Sweden; 4 Department of Pediatric Surgery, Karolinska University Hospital, Stockholm, Sweden; 5 Department of Pediatric Surgery, Queen Silvia’s Children’s Hospital, Gothenburg, Sweden; 6 Department of Pediatric Surgery, University Children’s Hospital, Uppsala, Sweden

**Keywords:** Sex, Surgical outcome, Hirschsprung disease

## Abstract

**Purpose:**

There are limited data to show how sex impacts the early clinical course of patients with Hirschsprung disease (HSCR). This study aimed to explore potential sex related disparities in the preoperative, surgical, and early postoperative course of HSCR patients.

**Methods:**

This retrospective study analyzed data of HSCR patients who underwent pull-through surgery at pediatric surgery centers in Sweden from July 1st, 2013, to June 30th, 2023. Male and female patients were compared regarding diagnostics, surgical treatment, unplanned procedures under general anesthesia or readmissions within 90 days after pull-through, and complications (Clavien-Madadi grade ≥ 3) up to 30 days after pull-through.

**Results:**

A total of 197 patients were included from four treating centers (158 males, 39 females). Females had a higher prevalence of familial disease (28.2% vs. 8.2%; *p* < 0.01) and *RET* gene mutations (15.4% vs. 2.5%; *p* = 0.02). No differences were observed in age at biopsy, need for re-biopsy, preoperative stoma rates, or age at diagnosis. Time from diagnosis to pull-through was longer in females (median 48.5 vs. 28 days; *p* = 0.02), but age at pull-through did not differ. No significant differences were found in postoperative hospital stay, severe complications within 30 days, nor unplanned procedures, HAEC, or readmissions within 90 days.

**Conclusion:**

The early clinical course of HSCR patients does not appear to be sex dependent. Although females had a longer interval from diagnosis to pull-through, their age at pull-through was comparable to males. As expected, a higher proportion of females reported familial disease and had a verified *RET*-mutation.

**Level of evidence:**

Level III.

**Supplementary Information:**

The online version contains supplementary material available at 10.1007/s00383-025-06213-5.

## Introduction

Hirschsprung disease (HSCR) is a congenital intestinal disorder that affects 1:5000 live births[1]. The anomaly is characterized by the absence of ganglion cells in the myenteric and submucosal plexuses of the distal colon, with various extent to proximal bowel[1]. HSCR can be classified by length of aganglionic segment as short-segment, long-segment, or total colonic aganglionosis (TCA). Short-segment disease, where aganglionosis is limited to the rectum and sigmoid colon, represents about 80% of cases. When the aganglionic segment extends beyond the sigmoid colon, the condition is classified as long-segment disease (15%) or TCA (5%), depending on the extent of involvement [2]. Treatment consists of early surgical resection of the aganglionic segment and the formation of an anastomosis between the normally innervated bowel and the anal canal, while preserving the sphincter complex[1, 3].

The overall ratio of male to female HSCR patients is 4:1[1]. However, this ratio approaches 1:1 in certain subgroups, such as familial HSCR, long-segment or TCA, and syndromic HSCR[4, 5]. Familial HSCR carries a significantly increased risk of recurrence, especially in long-segment disease, where the recurrence rate may reach 20–50%[6]. Increased segment lengths and particularly TCA is associated with more long-term complications, including persistent bowel dysfunction and recurrent Hirschsprung-associated enterocolitis (HAEC)[7]. Syndromic HSCR, seen in about 12% of cases, often leads to poorer functional outcomes due to cognitive impairments and genetic comorbidities, with a higher likelihood of requiring a permanent stoma[1, 8, 9]. Collectively, the decreased sex ratio of these subgroups may lead to a different composition of disease characteristics within the female HSCR population as compared to males.

For other pediatric surgical diagnoses, sex has been suggested as an independent predictor of outcome[10–12]. Differences in male and female HSCR patients have been observed in long-term outcomes such as fertility and sexual function[13–16]. Regarding the early clinical course, limited evidence indicates that males may experience more hospitalizations within the first year after pull-through, despite similar postoperative outcomes and no notable sex differences in preoperative symptoms, treatment approaches, or overall bowel function[17].

Although both male and female HSCR patients are typically included in studies, the disease's low prevalence and consequently small sample sizes often limit the ability to draw meaningful conclusions about sex-specific outcomes. In the few studies that do attempt sex-based analyses, the small overall cohorts, and particularly limited number of female participants, often lead to significant statistical uncertainty and risk of type II errors in results.

Considering the above, there is a continued need to assess potential sex-based differences in HSCR. The aim of this study was therefore to explore potential sex disparities in the preoperative, surgical, and early postoperative course of HSCR patients.

## Methods

### Study design

This was a retrospective observational study.

### Study population

All patients who had undergone pull-through at one of the pediatric surgery centers in Sweden from 1st of July 2013 to 30th of June 2023 were included. Patients were identified from the digital case record systems of participating sites.

### Treatment practice for HSCR patients across Sweden

The diagnostic workup for HSCR typically includes rectal suction biopsies and a contrast enema. At one participating center (Gothenburg), anorectal manometry has also routinely been performed as part of the diagnostic evaluation. Once HSCR is suspected, daily transanal irrigations are initiated and continued until definitive pull-through surgery. If transanal irrigations are chosen as the primary mode of preoperative management, caregivers receive training to perform them independently. After demonstrating competence, the patient is discharged and managed at home with ongoing access to specialist nurse support, ensuring safe and effective home care while awaiting surgery. In cases where transanal irrigations are inadequate, a temporary stoma may be created to decompress the bowel prior to reconstruction. Definitive pull-through is generally performed by two–to three months of age. The most performed procedures are transanal endorectal pull-through with either laparoscopic assistance or a subumbilical incision for biopsies. Routine genetic testing for HSCR is not performed nationwide in Sweden. Following the centralization of care 2018, patients are now managed at either the Stockholm or Lund centers. In Stockholm, genetic testing is offered to all patients and strongly recommended to those with a higher risk of mutations (e.g., familial disease, total colonic aganglionosis, or associated anomalies/syndromes), while Lund more frequently adopts a practice of universal genetic screening.

### Participating centers

The participating centers were: Karolinska University Hospital, Stockholm; Skåne University Hospital, Lund; University Children’s Hospital, Uppsala; and Queen Silvia's Children's Hospital, Gothenburg. Individual data collection was done by the patient’s treating site and all statistical analyses were performed at the coordinating site at Karolinska University Hospital.

### Data collection and measurements

Data were obtained retrospectively by review of electronic medical records. Demographic variables included sex, birth weight, gestational age, family history of HSCR, presence of other congenital anomalies and/or syndromes, genetic disorders, and place of birth. Gestational age was defined as: full-term (37–41 weeks), preterm (< 37 weeks), and post-term (≥ 42 weeks). Preoperative and surgical data recorded considered age at initial rectal biopsy, repeat rectal biopsy, age at histopathological diagnosis, requirement for stoma, extent of the aganglionic segment, surgical method, and duration of hospital stay. Unplanned hospital readmissions, unplanned surgical procedures under general anesthesia, and HAEC, within 90 days post-pull-through were assessed. Postoperative complications within 30 days were classified using the Clavien-Madadi system, considering only grade three or more. The occurrence of HAEC was determined from clinical notes, with an episode defined as a strong clinical suspicion by the attending physician leading to treatment with antibiotics.

### Statistical analysis

Statistical analysis was performed using SPSS (version 29.0.2.0) and R programming language (version 4.2.1) in RStudio (version 2022.07.01). Data wrangling was performed using dplyr, tables were created using tibble and gt, and diagrams were created using ggplot2 unless otherwise specified. Data are presented as percentages for categorical variables as well as median and range for numerical variables. Chi-square was used for comparative statistics of categorical data if applicable and Fisher’s exact test when assumptions of Chi-square were violated. The Mann–Whitney *U*-test was used for numerical data when comparing two independent groups. Missing data were reported but excluded from the analysis of numerical variables. Statistical significance was set at *p* ≤ 0.05.

### Ethics

This study has been approved by the Swedish Ethical Review Authority. All procedures are in accordance with the Helsinki declaration and its later amendments[18].

## Results

### Study population

A total of 197 HSCR-patients had undergone pull-through at one of the participating centers during the inclusion period (1st of July 2013 to 30th of June 2023). Of these, 158 (80.2%) were males and 39 (19.7%) females. Inclusion of patients from individual sites were as follows: Stockholm, 81; Uppsala, 26; Lund, 61; and Gothenburg, 29.

### Patient characteristics

Comparing males to females, no significant differences were found regarding gestational age, birth weight, associated malformations and/or syndromes, nor length of aganglionic segment (Table 1). Females had a higher reported prevalence of familial disease (28.2% vs. 8.2%; *p* = 0.002) and a higher proportion of verified *RET*-mutations (15.4% vs. 2.5%; *p* = 0.019).

**Table 1:** Demographic features

Patient characteristics	Male *n* = 158	Female *n* = 39	*p*-value
Gestational age, *n* (%)			0.461
Pre-term	15 (9.5)	1 (2.6)	
Term	124 (78.5)	32 (82.1)	
Post-term	8 (5.1)	3 (7.7)	
Unknown	11 (7)	3 (7.7)	
Birth weight, g, median (min–max)	3599 (1854–4750)	3554 (2540–4226)	0.370
Unknown, *n* (%)	14 (8.9)	4 (10.3)	
Familial disease, *n* (%)	13 (8.2)	11 (28.2)	**0.002**
*RET*-mutation, *n* (%)	4 (2.5)	6 (15.4)	**0.019**
Associated malformations and/or syndromes, *n* (%)	24 (15.2)	10 (25.6)	0.154
Trisomy 21	13 (8.2)	3 (7.7)	
Other syndrome	4 (2.5)	4 (10.3)	
Type of HSCR, *n* (%)			0.104
Rectosigmoid	136 (86.1)	28 (71.8)	
Long segment	17 (10.8)	5 (12.8)	
Total colonic aganglionosis	5 (3.2)	4 (10.3)	
Unknown	0	2 (5.1)	

Significant *p*-values ≤0.05 are in bold

### Outcomes

**Fig. 1:**
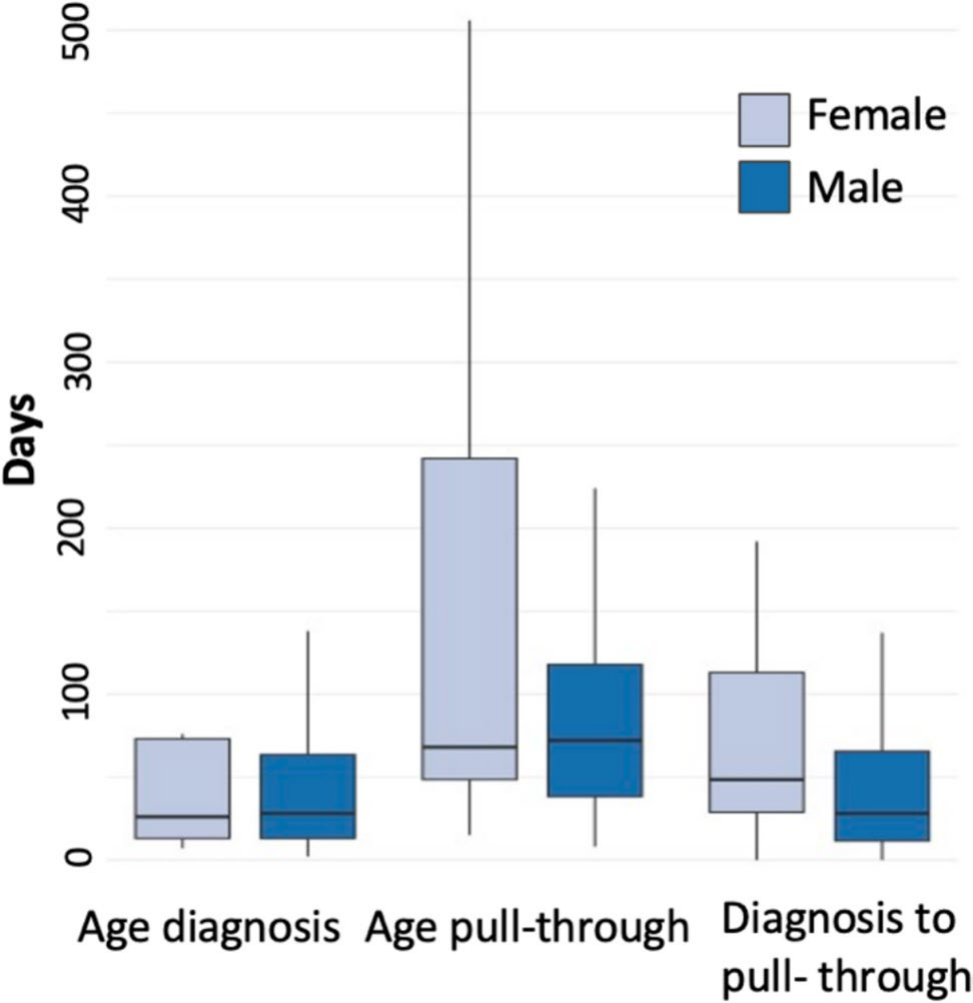
Age and time-related metrics by sex

Age at biopsy, need for re-biopsy and age at diagnosis did not differ between sexes (Table 2). Time from diagnosis to definitive pull-through was significantly longer in females (48.5 vs 28 days; *p* = 0.016) but age at pull-through and preoperative stoma rates were similar between groups. Comparing patients with rectosigmoid disease and long segment or TCA separately showed no differences in any of the variables in Table 2. In female patients with > 100 days from diagnosis to pull-through, seven out of ten had a primary stoma. Data concerning age at pull-through for females had a wider range than males with a significant right skew (Fig. 1). The most common surgical treatment in both groups was total transanal endorectal pull-through, followed by laparoscopically assisted endorectal pull-through. Two patients who first had a total transanal pull-through were mistakenly diagnosed with short-segment disease and eventually required revision surgeries.

**Table 2:** Diagnostics and surgical treatment

	Male *n* = 158	Female *n* = 39	*p*-value
Age at biopsy, days, median (min–max)	7 (1–1928)	8.5 (2–2000)	0.390
Re-biopsy, *n* (%)	37 (23.4)	6 (15.4)	0.202
Age at diagnosis, days, median (min–max)	28 (2–1985)	26 (7–2039)	0.994
Preoperative stoma, *n* (%)	31 (19.6)	10 (25.6)	0.388
Time from diagnosis to pull-through, days, median (min–max)	28 (0–1021)	48.5 (0–960)	**0.016**
Age at pull-through, days, median (min–max)	72 (8–2091)	68 (15–2231)	0.277
Surgical treatment *n* (%)			0.083
* Rectosigmoid and long segment disease*			
Total transanal endorectal pull-through, with or without subumbilical incision for biopsies	88 (55.7)	19 (48.7)	
Endorectal pull-through, with laparotomy	28 (17.7)	5 (12.8)	
Endorectal pull-through, laparoscopy assisted	33 (20.9)	10 (25.6)	
Endorectal pull-through, robot-assisted	1 (0.6)	0	
Duhamel (open)	0	1 (2.6)	
Unknown	3 (1.9)	0	
* Total colonic aganglionosis*			
Duhamel (open)	1 (0.6)	2 (5.1)	
Endorectal pull-through*	4 (2.5)	2 (5.1)	

*Including two patients initially misdiagnosed as rectosigmoid disease that later underwent revision

Significant *p*-values ≤0.05 is in bold

There were no sex-based differences in the postoperative outcomes (Table 3). Postoperative hospital stay was similar between males and females, five and six days respectively. There were no differences in severe complications (Clavien-Madadi ≥ 3) in the first 30 days following pull-through, nor unplanned readmissions, unplanned procedures under general anesthesia and HAEC requiring treatment with antibiotics up to 90 days post-pull-through. No complications grade IV or V were recorded during the time-period. One death was reported. The child was found deceased at home, and no definitive cause of death could be determined.

**Table 3:** Postoperative outcome

	Male *n* = 158	Female *n* = 39	*p*-value
Postoperative hospital stay, days, median (min–max)	5 (2–42)	6 (2–15)	0.819
Complications Clavien-Madadi (> 3) (30d), *n* (%)	12 (7.6)	3 (7.7)	1.00
IIIa	4 (2.5)	1 (2.6)	
IIIb	8 (5.1)	2 (5.1)	
HAEC (90d) *n* (%)	25 (15.8)	7 (17.9)	0.807
Unplanned readmission (90d) *n* (%)	26 (16.5)	9 (23.1)	0.361
Unplanned surgical procedure (90d) *n* (%)	23 (14.6)	5 (12.8)	1.00
Death *n* (%)	1 (0.6)	0	1.00

## Discussion

### Key findings

In this retrospective multi-center study, all male and female patients surgically treated for HSCR in Sweden over a 10-year period were compared by sex concerning the early clinical outcome, from initial diagnostics up to 90 days following pull-through. Females had a higher proportion of reported familial disease and verified *RET *mutation. Time from diagnosis to pull-through was significantly longer and more heterogenous in females, but age at diagnosis, age at pull-through and preoperative stoma rates did not differ. No sex-based differences within surgical treatment or initial postoperative outcome were found.

### Interpretation

Within this cohort, few sex-dependent outcomes were observed. Similar to results of previous studies an overall male–female ratio of 4:1 was observed, with a decreased sex-ratio in familial disease 1:0.9, *RET*-mutations 1:1.5, TCA 1:0.8, and syndromic HSCR 2.3:1 [1, 4, 5]. We hypothesized that the proportional increase of these features within the female cohort would impact the early clinical outcome as longer segments and syndromic HSCR is typically linked to poorer outcome. Interestingly, we found no significant differences in the diagnostic, surgical, or postoperative course apart from a longer time from diagnosis to pull-through in females of 48.5 days compared to 28 days in males. For other pediatric surgical diagnoses, sex has been connected more clearly to outcome. Stone et. al considered various pediatric surgical interventions and found female sex to be associated with lower post-operative morbidity yet longer post-operative hospital stay[10]. Other studies have suggested a higher in-hospital and 30-day post-discharge mortality for females undergoing congenital heart surgery and a higher resource utilization in male pediatric patients with acute sinusitis[11, 12].

A previous study on sex differences in HSCR, conducted in Lund with a smaller patient group, also reported minimal variation between male and female patients. Over a longer follow-up period, it was noted that males might have a higher rate of hospitalizations within the first year post-pull-through, even though preoperative symptoms, treatment methods, and overall bowel function were similar across sex and postoperative outcomes showed no significant differences[17].

Time from diagnosis to pull-through was longer in females, while conversely age at diagnosis and age at pull-through did not differ. A closer examination of the age-at-surgery data reveals greater variability among females, with a pronounced right skew as a possible explanation for the discrepancy. One interpretation could be that female patients represent a more heterogenous group. In our cohort, co-morbidities such as associated malformation or syndromes, preoperative stoma rates or length of segment were comparable between sexes and did not clearly account for the surgical delay observed in females. It is, however, noteworthy seven out of ten females with more than 100 days from diagnosis to pull-through had a preoperative stoma. Additionally, when comparing male and female patients with only rectosigmoid disease, there were no differences in time from diagnosis to pull-through. The Global Gender Gap Index (GGGI) ranks Sweden among the most gender-equal countries globally, suggesting that the observed difference is less likely to result from sex-based disparities in the allocation of healthcare resources[19].

Regardless, it is important to note that the observed delay in time to pull-through for females did not correspond with worse postoperative outcomes as no difference in postoperative hospital stay, severe complications, readmission rates or HAEC was observed.

### Limitations

The results of this study should be interpreted in the light of several limitations. Although it represents the largest study to date examining sex-based outcomes during the early clinical course, the cohort size remains limited. This may reduce statistical power and increase the risk of type II errors**.** The 10-year study period may introduce confounders, including changes in senior staff and protocols, evolving evidence, and shifts in treatment practices. Centralization of HSCR care in 2018 and the COVID-19 pandemic may have affected resource allocation during the study period, though likely without sex-specific impact. The retrospective design of the study may also impact the accuracy of the collected data. Moreover, since data collection was carried out by multiple individuals, there is a risk of inconsistent interpretation of the reviewed variables. It is important to note that genetic testing was not uniformly performed across all patients, and documentation regarding who underwent testing was inconsistent. This limits our ability to draw firm conclusions about the true prevalence of *RET*-mutations by sex, although testing was offered broadly and without sex-based restrictions, reducing the likelihood of systematic bias.

### Generalizability and clinical implications

This study provides a unique national overview of sex-based differences in the early clinical course of HSCR, including all surgically treated patients in Sweden over a 10-year period. Despite being the largest study of its kind, the rarity of HSCR and the limited cohort size still constrain generalizability, particularly to populations with different healthcare systems or sociocultural contexts.

Our findings suggest that sex does not significantly impact diagnostic, surgical, or short-term postoperative outcomes in HSCR, despite known differences in genetic and syndromic profiles. This implies that the current clinical management strategies and treatment guidelines remain broadly applicable to both males and females during the early clinical course of the disease. The lack of sex-based differences in diagnostic and perioperative outcome supports the notion that the basic therapeutic approach does not need to differ significantly between sexes in this period of care.

The observed delay from diagnosis to pull-through in females, though not reflected in worse outcomes, warrants further investigation. It raises the possibility of subtler sex-related differences in either clinical or systemic factors. These factors require further study to determine if results are driven by differences in symptom reporting, cohort characteristics, diagnostic practices, or even socio-cultural factors that could affect how healthcare providers perceive or manage male and female patients. Understanding these factors is an important part of refining our approach to early diagnosis and treatment, particularly if any sex-based disparities are contributing to delays in timely surgical intervention.

This study highlights the necessity of integrating sex as a significant variable in future clinical research. While sex differences were minimal in the early clinical outcomes, emerging evidence suggests that sex may play a more pronounced role in long-term outcomes, particularly regarding fertility, sexual function, and psychological well-being[13–16]. These factors may require tailored approaches to treatment and care, as sex-specific risks and needs may evolve over time. Incorporating sex as an ongoing consideration throughout the disease course will help ensure that HSCR patients receive individualized care that appropriately addresses their long-term risks.

This study emphasizes the importance of continued sex-based research in pediatric surgical conditions like HSCR, with the goal of improving risk stratification and providing a personalized approach to treatment. A more nuanced understanding of how sex influences not only early clinical outcomes but also long-term health outcomes will enable clinicians to optimize care strategies for both male and female patients in a holistic manner.

## Conclusion

The early clinical course of HSCR patients does not appear to be sex dependent. Although females had a longer interval from diagnosis to pull-through, their age at pull-through was comparable to males. As expected, a higher proportion of females reported familial disease and had a verified *RET*-mutation.

## Supplementary Information

Below is the link to the electronic supplementary material.

Supplementary file1 (DOCX 16 KB)

## Data Availability

No datasets were generated or analysed during the current study.

## Abbreviations

HSCR: Hirschsprung disease
TCA: Total colonic aganglionosis
HAEC: Hirschsprung-associated enterocolitis
