# Supplementary material for: Exploring potential sex differences in Hirschsprung disease: a national cohort study of diagnostic patterns and early postoperative outcome
Source: Pediatr Surg Int. 2025 Oct 3;41(1):309. doi: 10.1007/s00383-025-06213-5 (PMC12494626; doi:10.1007/s00383-025-06213-5)
Supplement: Supplementary file 1 — Supplementary file1 (DOCX 16 KB) [file 383_2025_6213_MOESM1_ESM.docx]

**Supplementary material: Exploring potential sex differences in Hirschsprung disease: a national cohort study of diagnostic patterns and early postoperative outcome**

**Supplementary Table 1: Complications to pull-through within 30 days**

|  | Male  n=12 | Female  n=3 |
| --- | --- | --- |
| Bowel obstruction/outlet obstruction* | 8 | 1 |
| Perforation | 1 | 1 |
| Seroma | 1 |  |
| Incisional hernia, wound dehiscence |  | 1 |
| Abscess | 1 |  |
| Urinary tract complication | 1 |  |

*One case of residual aganglionosis, two obstruction unspecified, four tight anastomoses, one outlet obstruction

**Supplementary Table 2: Reason for readmission within 90 days after pull-through**

|  | Male  n=26 | Female  n=9 |
| --- | --- | --- |
| Bowel obstruction/outlet obstruction | 10 | 4 |
| Hirschsprung-associated enterocolitis | 8 | 3 |
| Other suspected infection | 3 |  |
| Incisional hernia, wound dehiscence |  | 1 |
| Abscess | 1 |  |
| Anal bleeding pain after manipulation | 1 |  |
| Other | 3 | 1 |
